# Bluetooth 5.1: An Analysis of Direction Finding Capability for High-Precision Location Services

**DOI:** 10.3390/s21113589

**Published:** 2021-05-21

**Authors:** Giovanni Pau, Fabio Arena, Yonas Engida Gebremariam, Ilsun You

**Affiliations:** 1 Faculty of Engineering and Architecture, Kore University of Enna, 94100 Enna, Italy; giovanni.pau@unikore.it (G.P.); fabio.arena@unikore.it (F.A.); 2 Department of ICT Environmental Health System, Graduate School, Soonchunhyang University, Asan 31538, Korea; yonas.engidag@gmail.com; 3 Department of Information Security Engineering, Soonchunhyang University, Asan 31538, Korea

**Keywords:** Bluetooth 5.1, Bluetooth Low Energy, Bluetooth Direction Finding, indoor localization, asset traceability, Internet of Things

## Abstract

This paper presents an in-depth overview of the Bluetooth 5.1 Direction Finding standard’s potentials, thanks to enhancing the Bluetooth Low Energy (BLE) firmware. This improvement allows producers to create location applications based on the Angle of Departure (AoD) and the Angle of Arrival (AoA). Accordingly, it is conceivable to design proper Indoor Positioning Systems (IPS), for instance, for the traceability of resources, assets, and people. First of all, Radio Frequency (RF) radiogoniometry techniques, helpful in calculating AoA and AoD angles, are introduced in this paper. Subsequently, the topic relating to signal direction estimation is deepened. The Bluetooth Core Specification updates concerning version 5.1, both at the packet architecture and prototyping levels, are also reported. Some suitable platforms and development kits for running the new features are then presented, and some basic applications are illustrated. This paper’s final part allows ascertaining the improvement made by this new definition of BLE and possible future developments, especially concerning applications related to devices, assets, or people’s indoor localization. Some preliminary results gathered in a real evaluation scenario are also presented.

## 1. Introduction

Factories, warehouses, and manufacturing facilities increasingly use tags for real-time tracking of asset location [1,2]. The data are typically integrated into a suitable cloud-based Industrial Internet of Things (IIoT) inventory control system for remote asset tracking [3,4,5,6]. Aside from Near Field Communication (NFC), a problem emerges. Most asset tracking solutions depend on battery-operated tags, forcing power consumption as low as possible. Additionally, some solutions can be unreliable and inaccurate when used indoors. For instance, Global Positioning System (GPS) tags are unreliable indoors, particularly in steel and reinforced concrete buildings [7].

Moreover, logistics companies seek to enhance supply chain effectiveness through real-time monitoring of resources, while companies aim to improve productivity by monitoring staff and customer movements [8]. As a result, the demand for location services is growing [9]. A question arises: why are asset traceability and indoor localization necessary for the IIoT?

Advanced IIoT inventory control systems require real-time monitoring of assets from the cloud anywhere in the world [10,11]. Department stores that house high-value products and equipment may require location tags to be affixed for inventory control and anti-theft aid. This situation allows warehouse workers and automatic picking equipment to quickly and efficiently locate an item and prepare it for shipment. For inventory management, assets’ existence and location can be easily determined and detailed for periodic current status reports. This condition is a more reliable method of providing inventory status than manually reviewing shipping manifests that track inbound and outbound assets [12]. In addition to IIoT inventory management systems, real-time location tracking of assets is used in anti-theft systems. If an item in a warehouse is not scheduled for shipment, the IIoT system can alert security if found near an exit. Real-time asset tracking can also accelerate service and delivery in a period where next-day delivery is rapidly evolving towards same-day delivery expectations [13,14].

For volume asset tracking, the location tag must be affordable and have long battery life. NFC tags do not use batteries but require the receiver to be within 20 cm of the tag, limiting its usefulness. GPS trackers are not reliable indoors, as satellite tracking signals can be blocked, especially by steel and reinforced concrete structures [1]. A popular asset tracking solution is based on the location function of the Bluetooth beacon. This method tracks a tag’s position by comparing the strength of a reference signal encoded in the beacon message with the received signal’s strength. The beacon’s position is then triangulated using three or more receivers to approximate the beacon’s position. Nevertheless, this approach does not provide the accuracy required for inventory management systems. Additionally, location accuracy can be affected by changes in humidity and moving objects, such as forklifts, workers, and doors [15,16]. Although the Bluetooth Received Signal Strength Indicator (RSSI) can be employed to measure the range from a recognized position, frequently, this methodology is not accurate for purposes such as an Indoor Location System (ILS) and resources traceability [17,18,19]. It is required a reliable, cost-effective, and accurate, battery-powered solution for wireless asset tracking that can be used in indoor environments, also offering long battery life [20,21,22,23,24]. Nevertheless, bringing up-to-date Bluetooth spec suggests a higher well-defined solution to address this issue. Specifically, version 5.1 of the Bluetooth Core Specification [25,26], sold as “Bluetooth 5.1 Direction Finding”, [27] has supplemented the Angle of Departure (AoD) [28] and Angle of Arrival (AoA) [29,30] radiogoniometry functions, which greatly facilitate developers the task of accurately determining the location of Bluetooth communication. The AoA and AoD techniques will be described subsequently in this paper, explaining how, thanks to the Bluetooth Core Specification improvements, their implementation has been simplified. The platforms on which to realize the Direction Finding applications will then be presented.

Bluetooth 5.1 allows producers to develop Indoor Positioning System (IPS) [31] and resource monitoring solutions more swiftly. Precisely, the specification included in [25] states that a Constant Tone Extension (CTE) is supplemented to a BLE packet, thus allowing a receiver to obtain “IQ” data (i.e., information concerning the quadrature and phase needed to determine the location of a device) from the signal. It is possible to estimate that it could also be significantly easier to shape the rules to achieve IQ sampling employing the Host-Controlled Interface (HCI) to tune the sampling controller through this improvement. Nonetheless, it is necessary to note that obtaining IQ information is not yet an easy operation, as it requires the employment of a precisely sketched wireless microprocessor and antenna array. When the IQ information is available to calculate the transmitter’s position, it is still necessary to consider the multi-path reception, the polarization and signal propagation delays, the noise, and the jitter [32].

The spec presented in [27] asserts that the version 5.1 of Bluetooth, also known as Direction Finding, triangulates the position of a battery-powered tag, based on the phase shift of the signal acquired at two or more innumerable antennas. As an outcome, it is reasonable to within 1 m. Moreover, it is reasonable to perceive that it is a low-cost location tracking method that can be practiced reliably indoors while allowing years of operation on a single button battery. The Bluetooth Direction Finding spec [27] states that CTE is added to the standard Bluetooth advertizing package. It is helpful to remark that the CTE is a continuous tone sent on a calculated frequency equal to the 250 Hz Bluetooth frequency. Since the CTE is independent of standard Bluetooth message packets, it does not interfere with or delays these packets. This situation allows the receiving antennas to achieve continuous and uninterrupted correction in real-time, solving real-time position tracking [33].

This paper intends to describe the Bluetooth 5.1 Direction Finding standard’s capabilities, analyzing the details included in the specs [25] as well as other works in the literature. It is helpful to note that this paper differs from the latter, for instance, [20,34,35], since it principally aims to review all the details required for a comprehensive perception of the novelties introduced in Bluetooth 5.1 regarding the benefits of indoor localization and resource tracking. Unlike the works cited previously, this article does not propose a new approach based on Bluetooth 5.1 for indoor localization or empirical validation. After introducing all the technical features, this paper presents the steps to develop practical radiogoniometry applications with some suitable platforms and hardware. Moreover, this manuscript introduces a simple validation test through a development kit.

This paper is organized as follows. Section 2 presents an overview of radiogoniometry techniques related to Bluetooth 5.1. Section 3 analyzes the signal direction estimation concerning the use of antenna arrays for radiogoniometry. Section 4 introduces the specific updates included in Bluetooth 5.1 Core Specifications, while Section 5 proves the feasibility of developing useful radiogoniometry applications with reliable platforms and development kits, also presenting the preliminary results obtained in a real evaluation scenario. Finally, Section 6 concludes the paper.

## 2. RF Radiogoniometry Techniques

RSSI-based Radio Frequency (RF) radiogoniometry provides the approximate distance based on signal strength. By making multiple measurements of the distance from different points, it is possible to obtain higher precision. RSSI has the transcendent advantage of requiring just an individual antenna in the device, dropping the sophistication, expense, and dimension of antenna arrangements. The disadvantage is a shortage of accuracy, which is limited to 3–5 m [36]. Another well-known radiogoniometry procedure is acknowledged as Time of Arrival (ToA), i.e., the time interval between sending a radio signal from a transmitter to a far receiver. It is necessary to note that this approach also uses an individual antenna per appliance solely but requires that each device possess an exact synchronized clock, and this is the downside. Position accuracy for ToA systems can approach about 1 m [37].

**Figure 1:**
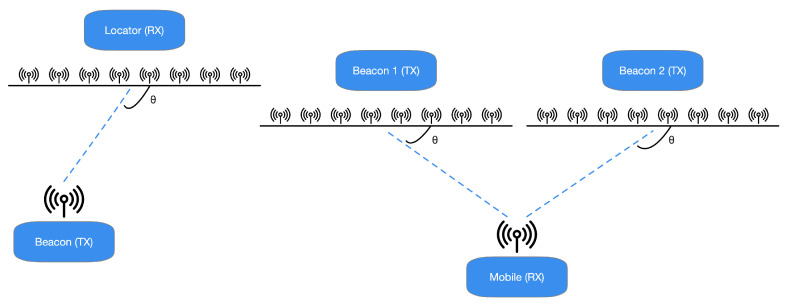
In the AoA radiogoniometry method (shown on the **left**), the resources transmit their position to an AoA identifier that gauges the signal’s arrival angle. On the contrary, practicing the AoD method (depicted on the **right**), beacons broadcast AoD data while a portable device collects the signals and estimates the point. Anyhow, the gathering device demands the computation capability to assess the place of the transmitter.

**Figure 2:**
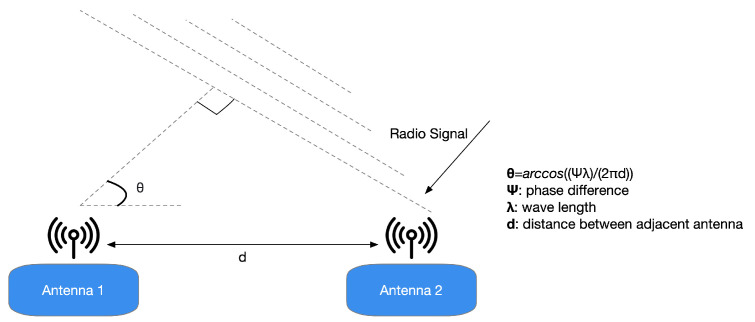
It is conceivable to estimate a radio signal’s arrival angle knowing specific information between neighboring antennas, such as each antenna’s distance, the wavelength, and the signal phase.

**Figure 3:**
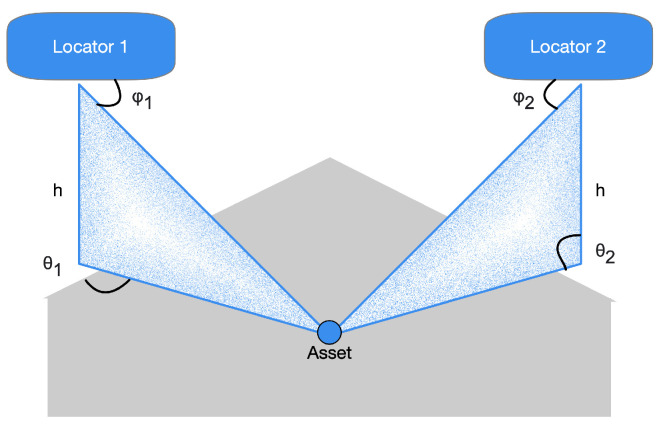
A transmitting resource’s three-dimensional position can be calculated by estimating the signals’ AoA in two fixed locators. If the locators’ absolute coordinates are known, it is also possible to calculate the absolute coordinates of the resource it transmits.

The specifications presented in [25] highlight that the Bluetooth Special Interest Group (SIG) has chosen to promote another radiogoniometry procedure focused on AoD and AoA. In the latter technique, a gathering device pursues the arrival angles for different things. Simultaneously, the AoD assesses its location practicing the angles of various beacons deployed in the environment to be analyzed and their positions, as depicted in Figure 1. It is possible to assert that the decision to incorporate a radiogoniometry peculiarity in Bluetooth 5.1 can be partially based on the circumstance that some organizations provide patented AoD and AoA techniques for BLE stocks. In detail, the analysis of [25] outlines that Bluetooth 5.1 allows developers to utilize RF radiogoniometry more efficiently thanks to the spec update to simplify the control of the IQ signal information (i.e., the data concerning in-phase and quadrature) of BLE packets. Consequently, thanks to this procedure, it is more straightforward to realize applications that include location services. For instance, it is possible to find that the AoA scheme is proper for ascertaining a BLE device transmitting. The transmitter sends radiogoniometry-enabled packets received by a multi-antenna “locator” using a single antenna. The receiver examines the IQ data included in the packets as they pass through all the array’s active antennas. This situation allows detecting the signal’s phase difference due to each antenna’s distance to the individual transmitting antenna. Then, the position processor handles data concerning the phase difference to ascertain the signals’ received angle and, consequently, the transmitter’s direction, as presented in Figure 2. Hence, the combination of the direction of the signals, calculated by several receivers, allows identifying a transmitter, as shown in Figure 3.

**Figure 4:**
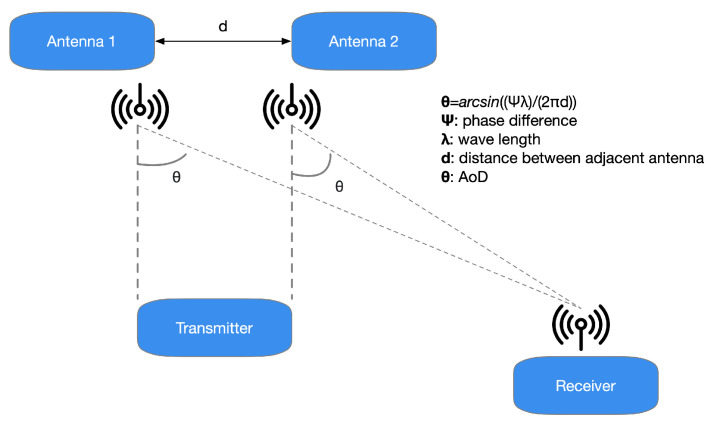
In the AoD technique, when every packet broadcasted by the antennas’ array comes at the antenna of the receiver, a phase-shifted regarding the preceding signal is appraised owing to the distinct length covered from the transmitter.

A reversed situation occurs in the case of the AoD method. In this different condition, the antenna array appliance transmits a data signal by its antennas. When every packet transmitted by the antennas’ array reaches the receiver’s antenna, the distinct distance covered from the transmitter ends in a phase-shifted regarding the prior signal (Figure 4).

The receiver antenna acquires IQ samples concerning the signal packets and reroutes them to the positioning system. The latter practices the obtained values to ascertain the angle of the collected signals. Consequently, the place of the transmitter is estimated. It is reasonable to perceive that this practice is suitable for indoor positioning applications. In these situations, a fixed reference point represents the transmitter, and, for instance, the receiver could be the consumer’s mobile device. It must be stated that, to propose a possible commercial solution for radiogoniometry, AoA is suitable for traceability of resources. In this circumstance, the transmitter is a movable component, for instance, a mere tag. At the same time, the locators, i.e., the receivers, are established points. Therefore, the specifications present in [25] outline possible advantages; for instance, the tag must transmit BLE 5.1 data utilizing an individual antenna (instead of an array). Furthermore, it is not necessary to perform computationally intensive algorithms to determine the position of the transmitter.

**Figure 5:**
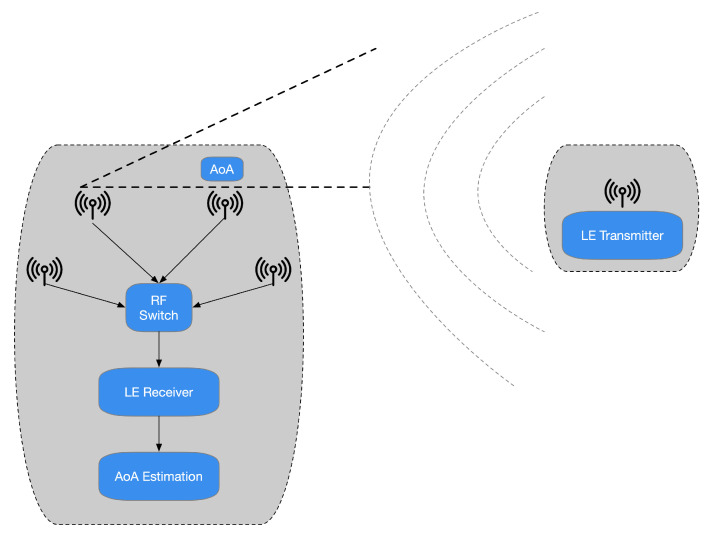
In an asset tracking system employing AoA radiogoniometry, the tag practices a unique antenna and a conventional BLE SoC to transmit Bluetooth 5.1 data, including the CTE. The principal calculations are then made at the locator of the system’s different antennas. Here, the locator’s signal data are sent to a locating scheme that operates the radiogoniometry techniques.

The examination of [25,26,27] raises some potential difficulties to be faced in the development of integrated solutions (i.e., System-on-a-chip—SoC). In this case, a further design challenge is represented by the fact that to contain costs BLE SoCs, in general, will have neither the ports for multiple antennas nor the ability to switching needed to process each antenna in the array systematically. As a result, an RF switch is required to connect the single antenna port of the BLE SoC to the different antennas in the array and switch from one antenna to another to collect IQ data from each of them (as depicted in Figure 5). The receiver (or locator) demands an antennas’ array intending to identify the signal’s phase difference through the IQ information, which caused the range variation between each antenna in the array and the unique broadcasting one. The AoA or AoD is discovered through the difference among the phase angle of every antenna.

Generally, antennas belong to three classes: Uniform Rectangular Array (URA) [38], Uniform Linear Array (ULA) [39], and Uniform Circular Array (UCA) [40]. The antenna array scheme demands much knowledge. Consequently, the developers prefer to assign the task of configuring the optimal array to an expert and drawing up a bill of materials for large volume production, as already discussed. The requirement for antenna arrays, supplementary memory, matched processors, and antenna administration increase the sophistication of the receiver side of the resource tracking method and cost and energy consumption [41]. The positive perspective is that the receivers generally remain in fixed positions so that the mains could power them. For most solutions, a relatively small number of devices will be demanded than the number of tags.

**Figure 6:**
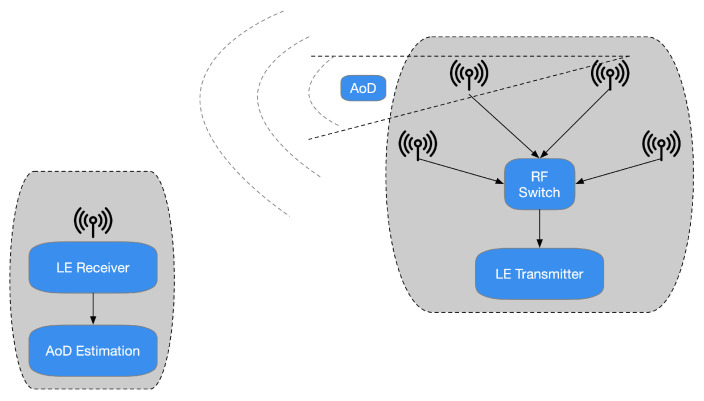
In an IPS system of AoD radiogoniometry, set beacons practice arrays of the antenna to transmit BLE 5.1 data with CTE. The foremost calculations take place in the mobile device, for instance, in a consumer’s smartphone.

AoD developments are a bit further complicated. In this situation, the antenna array is embedded in the transmitter. The receiver executes IQ sampling, practicing measurements of its antenna. The transmitter antenna design of the remote transmitter is employed to assign various measurements to each specific antenna. On the contrary, the specification noted in [25] states that, in the AoD development, set receiver beacons expect a BLE 5.1 transceiver, multiplied antennas, and an RF switch to broadcast the beacon data. However, a powerful processor and an extra memory demanded in AoA implementations are not required since no signal examination is conducted on this way of the connection. Nevertheless, although the portable receiver requires a single antenna solely, it does not demand both software and hardware to achieve radiogoniometry calculations, as depicted in Figure 6. For instance, it is possible to assume that, for IPS purposes, the receiver could be a BLE 5.1-compliant [25] smartphone [42] with good memory and CPU support to achieve the assignment.

## 3. Signal Direction Estimation

**Figure 7:**
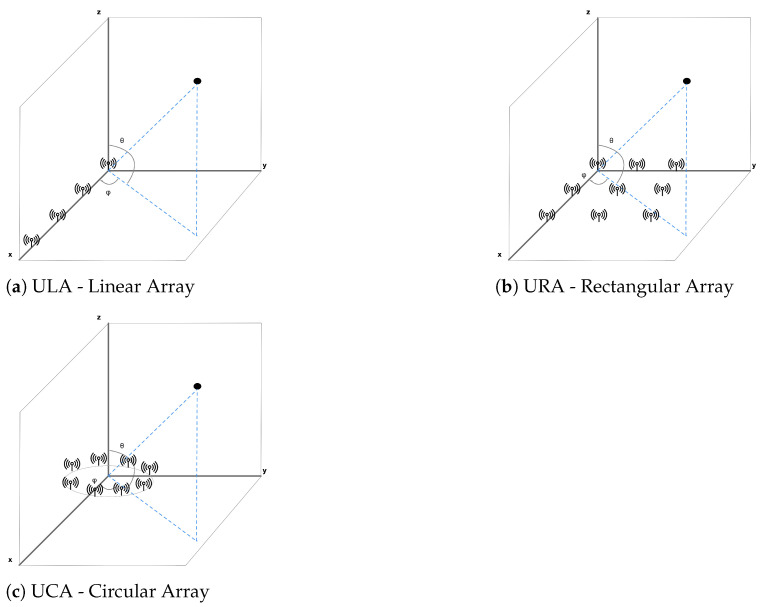
AoA and AoD radiogoniometry techniques require antenna arrays, whose common shapes are circular, rectangular, and linear. Every model of the array allows obtaining information concerning elevation and azimuth. Anyhow, rectangular and circular ones afford more further steady azimuth information

Antenna arrays for radiogoniometry are ordinarily classified into three types: ULA, UCA, and URA. More in detail, the rectangular and circular ones are two-dimensional, while the linear array is one-dimensional. The ULA is the simplest to sketch and actualize; however, it has the disadvantage of determining the azimuth angle considering that the traced device continuously passes on the equivalent flat. On the contrary, accuracy would be compromised if this situation does not occur. UCAs and URAs allow reliably estimating both elevation angles and azimuth (Figure 7).

Designing an array of antennas for radiogoniometry is no small task. For instance, when the antennas are arranged in an array, interference happens for mutual coupling. As a consequence, calculation techniques regularly expect default array acknowledgments to account for these effects. For instance, a standard algorithm assumes that the array is made up of two identical subarrays. Fortunately, for those not familiar with antennas, antenna arrays with defined characteristics are commercially available [43].

A helpful antenna array will ensure the collection of accurate IQ samples. However, the “unprocessed” data are inadequate to estimate the signal’s route. They need to be treated, taking into account multi-path reception, polarization, signal spread delays, jitter, and noise. Since radio frequency radiogoniometry is not a brand-new research domain, numerous analytical solutions evaluate the IQ samples’ arrival angle. The arrival angle assessment, or the reckoning for the starting angle, of a transmitted signal (narrowband) that comes at the gathering array, is considerably manageable. Consequently, also the mathematical computations needed to resolve it are concise.

Basically, as shown in [44], given a dataset of IQ samples for every antenna within the array, the popular methods initially determine an “*x*” data vector taking into account the Equation (1), assuming that the signals are sinusoidal (narrowband) phase-shifted and scaled:

(1) x(t)=r(θ)S(t)+n(t)

in which “*r*” is an analytical description of the antenna array (i.e., the “guide vector”), “*S*” represents the arriving signal, and “*n*” is taking into account for noise. Thus, *x* is employed to produce the covariance matrix of the IQ sample “$R_{xx}$” practicing the Equation (2):

(2) $$R_{xx}\approx \frac{1}{N}\sum_{t=1}^{N}x\left(t\right)x^{H}t$$

This matrix is next utilized as an entry to the principal predicting method. Multiple Signal Classification (MUSIC) [45] represents a traditional approach for frequency calculation and radiogoniometry. This solution practices the eigenvectors’ disengagement and the covariance matrix’s eigenvalues to estimate arrival angle, taking into account the signal and noise subspaces’ characteristics. Then, the following Equation (3) is adopted:

(3) $$R_{xx}\approx VAV^{-1}$$

where “*A*” represents a diagonal matrix comprising the eigenvalues and “*V*” is another matrix including the analogous eigenvectors. Subsequently, *V* can be separated. Hence, it can be practiced in an equation that produces a sort of pseudo spectrum, causing a peak at the arrival angle of the obtained signal, namely:

(4) $$P\left(\theta \right)=\frac{1}{r^{H}\left(\theta \right)VV^{H}r\left(\theta \right)}$$

**Figure 8:**
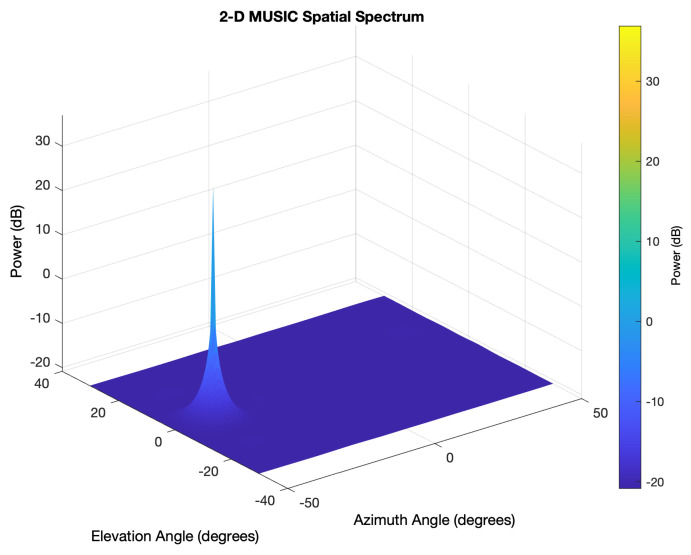
The MUSIC method practices IQ samples to generate a pseudo power spectrum, in which the tip identifies the location of the broadcasting device. The graph depicted in this Figure exhibits a 2D pseudo spectrum, in which the transmitting device is at an azimuth angle of −37 degrees and an elevation angle of 0 degrees.

The resulting spectrum, obtained through Matlab script [46], is depicted in Figure 8, where the peak occurs in the way from which the broadcasted signal reaches the destination.

## 4. Bluetooth 5.1 Updates

The specification introduced in [25,26,27] highlights that Bluetooth 5.1 requires modifications to the radio frequency “stack”, i.e., the software protocol. Moreover, also hardware enhancements are expected depending on the chip manufacturer. It is beneficial to heed that, as the main feature, the updated protocol attaches a CTE to every Bluetooth data employed for radiogoniometry. Otherwise, packets remain unchanged to be practiced for conventional BLE transmission [25,26]. The analysis of [25] denotes that CTE is not modulated and transmitted at 250 kHz or, seldom, more than 500 kHz when employing the more effective throughput method of Bluetooth. Its continuance is between 16 and 160 μs. More in detail, as introduced in [25], CTE is composed of an “unwhitened” series of “1” communicated large adequate to permit the receiver to obtain the IQ information without modulation’s influences. As the CTE is transferred at the end, the Cyclic Redundancy Check (CRC) of the packet is not influenced.

**Figure 9:**
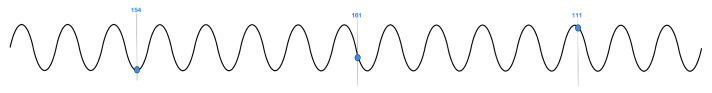
A single transmitter signal shows a different phase when it reaches the antennas at different distances from the source.

Another notable enhancement to the spec [25] greatly simplifies protocol configuration for IQ sampling. This arrangement comprises arranging both the antenna switched and the sample timing, i.e., two fundamental values for the position estimation accuracy. Ordinarily, although several IQ sampling timing arrangements can be practiced, an IQ representation is registered every 1 or 2 μs inside the associate time per antenna. Moreover, the outcomes are reported in the Random Access Memory (RAM) of the BLE SoC. Figure 9 shows how the received signal phase varies as different array antennas sample it.

Registering IQ samples denotes only the initial action in developing a usage that comprises a location service. Developers must invent or choose the optimal array of antennas for the locators and beacons to accomplish the task. Besides, they must become familiar with the complicated techniques demanded to make radiogoniometry calculations.

**Figure 10:**
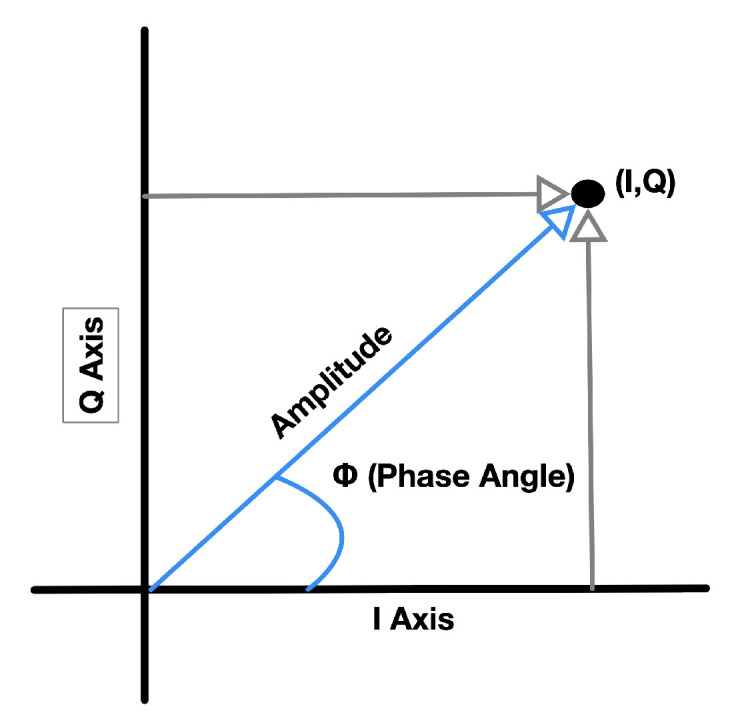
In radiogoniometry applications, the receiving BLE device takes IQ samples of phase angle and amplitude throughout the CTE part of a BLE package for every array’s antenna. Then, this information can be reproduced as cartesian coordinates (I, Q).

As introduced in [25], unique BLE 5.1 packets incorporate a CTE consisting of digital “1 s” to guarantee that the antenna receives a constant frequency for this part of the signal (rather than the changed frequency customarily employed to transmit BLE data). Besides, it is helpful to note that this information string is not “whitened”. Consequently, an adequately developed BLE radio that receives CTE information takes IQ examples throughout the CTE period. More in detail, a unique IQ sample is composed of the signal phase angle and amplitude expressed as cartesian coordinates (Figure 10).

**Figure 11:**
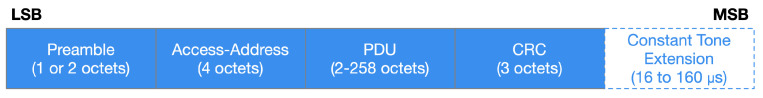
Structure of a Bluetooth 5.1 packet with the duration and position of the CTE. Associated apps add CTE to conventional packets, while those without a connection use an advertizing packet.

Bluetooth Core Specification v5.1 specifies the BLE controller modifications that acknowledge AoD and AoA methods to practice connectionless or connected (“paired”) transmissions [25,26]. Commonly, the AoA technique is employed for connected purposes (for instance, resource traceability), while the AoD technique is practiced with connectionless scenarios, such as IPS. Thus, as clearly described in [25], connected radiogoniometry adopts conventional BLE 5.1 packets, if anything, by introducing CTE at termination. On the other hand, radiogoniometry without connection practices a CTE appended to the periodical Bluetooth advertizing packets (Figure 11). In both scenarios, with and without connection, the maker has to deliver several arrangement actions to start the CTE on the transmitter and the IQ sampling on the receiver. The exact process depends on the choice between AoA or AoD-based applications.

## 5. Practical Use of Bluetooth 5.1

Running radiogoniometry algorithms involves many calculations and requires much RAM and Flash memory. Consequently, all commercial solutions have to consider these requirements. The devices implemented must be suitable for both transmitting and receiving in a Bluetooth radiogoniometry application. Each of them needs to support CTE transmission and acquire IQ samples due to profile-level data guidance that specifies the transmitter antenna layout. In theory, these devices can likewise deliver the complicated computations required to determine the incoming radio signal’s angle of incidence and, from this, the transceiver’s position.

Nordic Semiconductor, Silicon Labs, and Dialog Semiconductor are focusing their architectures on AoD and AoA solutions that broadcast CTE, collect these data and make IQ sampling. Consequently, the maker has to choose which means (i.e., firmware and hardware of the localization engine) will perform the actual radiogoniometry calculations. Nonetheless, things will likely change soon as suppliers release advanced radiogoniometry solutions.

For instance, each company offers development tools that support a tag’s prototyping in an AoA resource tracking scenario. The growing method typically matches that of a typical low-power wireless equipment. Moreover, for testing, a development kit must include an entirely operative serial transceiver built on the BLE 5.1 and other peripherals. The prototyping board can be attached to a computer that entertains a proper Integrated Development Environment (IDE) and the Software Development Kit (SDK) of the chip vendor to enable application development. In this paper, three possible examples of commercial solutions produced by distinct industry leaders have been identified.

Dialog Semiconductor promotes the employment of Bluetooth 5.1 with the development kit DA14695-00HQDEVKT-P-ND [47]. This assortment comprises the main motherboard, an offspring card containing the Bluetooth 5.1 SoC DA14695, and wiring for connection with a computer. Silicon Labs proposes the Wireless Gecko SLWSTK6006A kit [48] that encompasses six daughter cards based on the SoC Bluetooth 5.1 EFR32BG21 that allow the prototyping of a traceability system of resources with multiple tags. Nordic Semiconductor proposes the nRF52840-DK [49] developed with nRF52840 SoC, which is thoroughly agreeable with the SoC Bluetooth 5.1 nRF52811.

A comparison of the previously mentioned prototyping boards is shown in Table 1.

**Figure 12:**
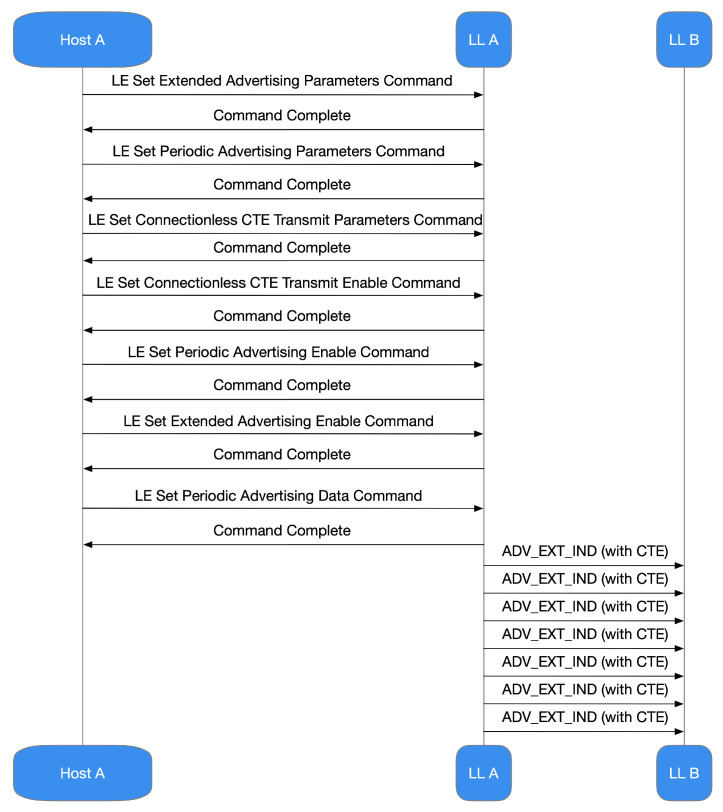
Actions concerning controller start-up delivered by the appliance for connectionless situations, i.e., those commonly practiced by AoD.

Bluetooth 5.1 does not broadcast data with CTE, nor does IQ sampling by default. It is up to the developer to set up the architecture to include these characteristics employing specific development instruments. The IDE and SDK concede the Host-Controlled Interface (HCI) management. Consequently, the host can practice it to set up the controller to make the CTE and IQ sampling. It is valuable to remark that, for connectionless situations (i.e., the model commonly practiced by AoD), the device delivers the subsequent startup stages of the controller (Figure 12):

1. configuring extended advertizing;
2. configuration of periodic advertizing;
3. configuration of the CTE transmission;
4. enabling CTE advertizing;
5. enabling periodic advertizing;
6. enabling extended advertizing;
7. setting of the advertizing data.

Scanning devices sketched to obtain CTE information and practice IQ samples transferred by the sender must be implemented in the following process:

1. set up the widespread scan
2. begin the widespread scan;
3. synchronize with the collected periodical advertizing packets;
4. let IQ sampling without connection.

In connected situations in which AoA is commonly practiced, both the devices (Master and Slave) expect the other device to transmit CTE data. In these cases, appeals are executed by transmitting a specific CTE Link Layer (LL) package comprising a group of peculiarities that set up the CTE formulation. If the distant device cannot sustain CTE, it will notify the local one, and the latter will not transmit additional CTE calls practicing the connection in progress. More in detail, the device making the request proceeds:

1. through the set up of reception parameters of the CTE in the controller;
2. through the enabling of CTE applications in the controller;
3. through the acquisition and management of IQ statements;
4. through maiming the communication of the CTE request when it is no longer needed.

The responding device proceeds:

1. through the set up of transmission parameters of the CTE in the controller;
2. through the enabling of CTE replies in the controller;
3. through the acquisition and reply to CTE LL inquiries from the other appliance.

In the Bluetooth 5.1 spec [25,26], the HCI includes a novel method, “LE Read Antenna Information”, which enables the device to gather data on the antennas held by its controller. So, it is helpful to perceive that when IQ sampling is achieved through an antenna array, every obtained sample need to be assigned to a definite antenna. Suddenly, the sampling has to be accomplished systematically. Using a model defined in the HCI arrangement rules and compliance with stringent timing commands facilitates this methodical procedure. The process these regulations are exercized and how each device practices them is strictly related to the employment, i.e., if it practices AoD or AoA and if the appliance is broadcasting or gathering. For instance, a transmitting device equipped with a unique antenna transmits consecutive information incorporating CTE. However, IQ sampling is regularly conducted by the acquiring device, regardless of the antennas it uses.

**Figure 13:**
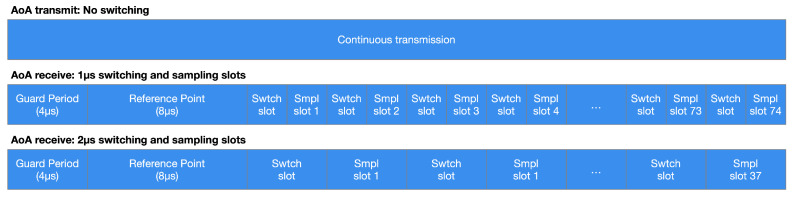
This example represents 1 μs and 2 μs timing switching and sampling in an AoA context. The broadcasting device, equipped with a unique antenna, continuously sends data with CTE while the receiving one performs IQ sampling according to a switching and sampling sequence.

The period designated for CTE management is separated within an opening guard period of 4 μs, a reference period of 8 μs, and then into a series of switch slots, sample slots, or combinations of them (Figure 13). During the reporting period, no antenna switching happens, and eight IQ samples are collected. The device could employ the reference samples to determine the signal frequency and deduce the wavelength, improving the accuracy of the angle calculations.

### Preliminary Results

In a real scenario, there could be various techniques for determining a tag’s location through Bluetooth. To this end, a measurement scenario, utilizing several boards included in the SLWSTK6006A starter kit [48] and configured following the steps mentioned above (Figure 12), was realized to evaluate the benefits introduced by Bluetooth 5.1 concerning indoor positioning. A laboratory, about 25 × 15 m wide, represented the reference scenario, composed of pillars, walls, human movements, tables, chairs, devices, and lamps. As stated previously, several receivers could be expected to practice position estimating methods and to deliver position finding computations. Anyhow, direction-finding can be obtained employing just one receiver. Nonetheless, to evaluate the position, it is clear that many directions (not just one) are expected to employ geometric techniques. Consequently, four anchors, i.e., receivers in the positioning system, were taken into account to reach the area of the reference scenario.

It has not been feasible to simultaneously practice four separate antenna arrays, considering the limitations of the test’s hardware appliances. On the contrary, a 4 × 4 URA, installed at 2 m height, was adopted, switching its position in diverse points within the reference scenario. The transmitter, situated at 1.2 m height, was the corresponding practice in the direction-finding estimations. Eight distinct locations in the laboratory were taken into account, and 32 different estimations were evaluated. It is helpful to note that the estimation method was commensurate in the direction-finding measures, except that the receivers’ adjustment was fixed.

For simplicity, a hybrid solution based on AOA and RSSI was assumed to evaluate the transmitter’s location, thus avoiding using more complex hardware. More in detail, the position parameter was represented in a 2-dimensional coordinates approach, aiming at building a geometric area of the reference scenario. Consequently, this coordinates arrangement, scaled twice the right system in meters, was employed as the global one, designing the transmitter’s location within the laboratory. In the considered geometric area, four local axes, matching with the receiving points, were acknowledged. In these cases, the x-axis of the local coordinates system was established perpendicular to the 4 × 4 URA, facing the stating point. Besides, the direction matched with Φ = 0°, i.e., equal to the coordinates utilized for direction-finding determinations. Virtually, from receiving points in the considered geometric area, it was possible to outline paths denoting intersections among the location of receivers and the reference point. Consequently, these paths could be turned based on the approximated angle of arrival at every location. The approximated range practicing the value of RSSI compelled the lines, rising in four positions. Then, the derivation of the geometric center of the positions allowed for getting the expected position.

In the considered evaluation scenario concerning position finding determinations, the impact of computational calculations was not taken into account. On the contrary, the main aim focused on achieving sub-meter error precision, practicing 48 Bluetooth packets for every eight locations. Hence, an amount of 16 positions were determined on each transmitting site. The obtained results are shown in Table 2. It is useful to note that Δm denotes the Euclidean range connecting the real position and the approximated one. Consequently, the results represented in Table 2 resemble the average distance error of 16 calculations for every location. The total average distance error was 0.7 m, i.e., a good result concerning the sub-meter precision purpose.

## 6. Conclusions

This paper has bestowed an in-depth overview of Direction Finding capability for high-precision location services. The analysis carried out showed that the Core Specification amendment assumed in Bluetooth 5.1 improves IQ information management. The latter can be practiced to support RF radiogoniometry approaches that measure the AoD or AoA of BLE wireless communication and then employ this knowledge to assess a transmitter’s location. Nevertheless, it is essential to remark on two concepts. The techniques can be utilized as a background in working position finding purposes, for instance, IPS and asset tracking, to name a few. However, their correctness can be rigorously related to a well-sketched antenna array, a certified wireless radiogoniometry method, CPU resources, and adequate memory to achieve complicated calculations. Anyhow, the availability of wholesale Bluetooth 5.1 Direction Finding architectures, localization-intended firmware, and antenna arrays could support planners to design employment aiming at localization services with centimeter precision.

An evaluation scenario regarding position finding determinations has been considered in this paper to achieve sub-meter error precision. The obtained results showed that the total average distance error was about 0.7 m, representing a reliable result regarding the sub-meter precision target. The goal of the simple test implemented in this paper was not to achieve an accuracy of the centimeter order, although Bluetooth 5.1 is theoretically able to achieve it, but below 1 m.

It is reasonable to conclude that the enhancements proffered to version 5.1 of the Bluetooth Core Specification produce the new information expected by radiogoniometry exercizing IQ sampling and CTE. The spec practices asserted manufacturing methods to ascertain the signal’s path. Moreover, it regulates interfaces, arrangements, and intercommunications. There is an extra benefit ascertained by the evidence that reasonable radiogoniometry is interoperable with all semiconductor vendors, which, in turn, offers application solutions for Bluetooth. Manufactures have been receptive to propose hardware, software, IDEs, and SDKs that permit developers to immediately comprehend how to set up systems that catch the benefit of Bluetooth radiogoniometry. Commercial IPS and radiogoniometry solutions demand considerable expertise, especially in treating firmware and antenna arrays for localization engines. However, future Bluetooth radiogoniometry profiles promise to simplify this challenge further.

## Figures and Tables

**Table 1:** Prototyping boards comparison.

Board	MCU	Memory	Output Power	Protocol Stack	Frequency Band	Interfaces	UI
DA14695-00HQDEVKT-P [47]	Cortex-M33F, Cortex-M0+	512 kB	6 dBm	Bluetooth	2.4 GHz	I/O, USB	LEDs, LCD Screen
SLWSTK6006A [48]	Cortex-M33	1024 kB	10 dBm	Bluetooth	2.4 GHz	I/O, USB	Buttons, LEDs, LCD Screen
nRF52840 DK [49]	Cortex-M4	64 MB	8 dBm	Bluetooth, Bluetooth Mesh, Thread, ZigBee	2.4 GHz	I/O, USB	Buttons, LEDs,

**Table 2:** Error regarding average distance.

Position	Average Δm
1	1.17 m
2	0.78 m
3	0.62 m
4	0.32 m
5	0.29 m
6	0.44 m
7	0.46 m
8	1.52 m
Total average Δm: 0.7 m	
